# The TGEV Membrane Protein Interacts with HSC70 To Direct Virus Internalization through Clathrin-Mediated Endocytosis

**DOI:** 10.1128/jvi.00128-23

**Published:** 2023-03-28

**Authors:** Zhaoyang Ji, Hui Dong, Ruixue Jiao, Xiaoyuan Zhu, Hongyan Shi, Jianfei Chen, Da Shi, Jianbo Liu, Zhaoyang Jing, Jialin Zhang, Xiaobo Wang, Dandan Ye, Jiyu Zhang, Xin Zhang, Li Feng

**Affiliations:** a State Key Laboratory of Veterinary Biotechnology, Harbin Veterinary Research Institute, Chinese Academy of Agricultural Sciences, Harbin, China; b Molecular Biology (Gembloux Agro-Bio Tech), University of Liège, Liège, Belgium; Loyola University Chicago—Health Sciences Campus

**Keywords:** transmissible gastroenteritis virus, membrane protein, HSC70, internalization, clathrin-mediated endocytosis

## Abstract

Coronavirus membrane protein is a major component of the viral envelope and plays a central role in the viral life cycle. Studies of the coronavirus membrane protein (M) have mainly focused on its role in viral assembly and budding, but whether M protein is involved in the initial stage of viral replication remains unclear. In this study, eight proteins in transmissible gastroenteritis virus (TGEV)-infected cells coimmunoprecipitated with monoclonal antibodies (MAb) against M protein in PK-15 cells, heat shock cognate protein 70 (HSC70), and clathrin were identified by matrix-assisted laser desorption ionization–tandem time of flight mass spectrometry (MALDI-TOF MS). Further studies demonstrated that HSC70 and TGEV M colocalized on the cell surface in early stages of TGEV infection; specifically, HSC70 bound M protein through its substrate-binding domain (SBD) and preincubation of TGEV with anti-M serum to block the interaction of M and HSC70 reduced the internalization of TGEV, thus demonstrating that the M-HSC70 interaction mediates the internalization of TGEV. Remarkably, the process of internalization was dependent on clathrin-mediated endocytosis (CME) in PK-15 cells. Furthermore, inhibition of the ATPase activity of HSC70 reduced the efficiency of CME. Collectively, our results indicated that HSC70 is a newly identified host factor involved in TGEV infection. Taken together, our findings clearly illustrate a novel role for TGEV M protein in the viral life cycle and present a unique strategy used by HSC70 to promote TGEV infection in which the interaction with M protein directs viral internalization. These studies provide new insights into the life cycle of coronaviruses.

**IMPORTANCE** TGEV is the causative agent of porcine diarrhea, a viral disease that economically affects the pig industry in many countries. However, the molecular mechanisms underlying viral replication remain incompletely understood. Here, we provide evidence of a previously undescribed role of M protein in viral replication during early stages. We also identified HSC70 as a new host factor affecting TGEV infection. We demonstrate that the interaction between M and HSC70 directs TGEV internalization in a manner dependent on CME, thus revealing a novel mechanism for TGEV replication. We believe that this study may change our understanding of the first steps of infection of cells with coronavirus. This study should facilitate the development of anti-TGEV therapeutic agents by targeting the host factors and may provide a new strategy for the control of porcine diarrhea.

## INTRODUCTION

Coronaviruses (CoVs) are spherical, enveloped RNA viruses that cause enteric, respiratory, and generalized diseases in many animal species, including humans (1, 2). CoVs can be divided into four genera (*Alphacoronavirus*, *Betacoronavirus*, *Gammacoronavirus*, and *Deltacoronavirus*), and transmissible gastroenteritis virus (TGEV) is an alphacoronavirus. Its genome is about 28.5 kb in length (3, 4) and encodes four structural proteins: the spike (S), membrane (M), nucleocapsid (N), and envelope (E) proteins. TGEV causes vomiting, watery diarrhea, dehydration, and body weight loss, and without adequate lactogenic antibody protection, the mortality rate in young piglets after TGEV infection is 70% to 100% (5, 6), resulting in substantial economic losses worldwide (7). TGEV was first identified in 1946 in the United States (8). It is currently an important etiological agent of disease in swine, causing the deaths of millions of piglets.

The first step in the viral infection process is its entry into cells. For CoVs, the process is initiated by the interaction between the spike protein (S) and its receptor(s), which is distributed in the cell membrane (9). It then fuses with the plasma membrane to enter the cell. The protein domains exposed on the viral surface usually play an important role during infection by binding to their cellular receptors, facilitating the process of cell fusion or the viral interaction with elements of the host immune system. The CoV M protein is the most abundant protein in the envelope of the virus (10). It contains three major regions: a small extracellular domain (ectodomain), a transmembrane domain, and a large carboxy-terminal domain (endodomain) (11). However, few reports have described the involvement of M in the entry process of CoVs. The signal peptide of the M protein is located at amino acids (aa) 1 to 16 (12). In contrast to S protein, M protein is generally considered to be involved in viral assembly and budding. Notably, the TGEV M protein adopts two topologies in the envelope, the Nexo-Cendo topology (with the amino terminus exposed to the virion surface) and the Nexo-Cexo topology (both the amino and carboxyl termini are exposed to the virion surface), and in a significant proportion of TGEV M protein molecules, the carboxy-terminal domain is exposed on the external surface of the virion (13, 14), suggesting that the TGEV M protein may be involved in the early stage of viral replication. Notably, the M protein of human CoV NL63 (HCoV-NL63) mediates viral attachment (15), suggesting that the CoV M protein is responsible for CoV entry, at least to some extent. Therefore, the role of the TGEV M protein in the early stage of virus replication should be identified.

Heat shock cognate protein 70 (HSC70) is a major molecular chaperone protein and a member of the heat shock proteins. Previous studies have shown that HSC70 is a cytosolic protein, and increasing evidence indicates the presence of HSC70 on the cell surface (16). HSC70 contains three domains: an amino-terminal ATPase domain (also known as the ATP-binding domain; aa 1 to 384), the substrate-binding domain (SBD) (aa 385 to 543), and the carboxy-terminal domain (also known as the lid domain; aa 544 to 646) (17, 18). HSC70 plays a major role in protein quality control, including in the refolding of misfolded proteins and the regulation of protein translocation. It also has many distinct functions in the prevention of protein aggregation and the disassembly of the clathrin complex (19, 20). HSC70 is also reported to participate in viral infection and is crucial to the infection processes of several viruses. For example, HSC70 interacts with the 2A protease of enterovirus A71 (EV-A71) to regulate its infection (21). HSC70 has been shown to be a viral receptor essential for rotaviral entry into cells (22), and recent studies have shown that HSC70 is required for infection by infectious bursal disease virus (23). HSC70 is also involved in the disassembly of cucumber necrosis virus particles (24). Therefore, HSC70 is involved in regulating the life cycles of various viruses (25).

In this study, we used coimmunoprecipitation (co-IP) and mass spectrometry (MS) to identify HSC70 as an M-interacting protein and demonstrated that this interaction played an important role in the internalization of TGEV. We also showed that the HSC70 SBD domain interacted with the clathrin heavy chain (CLHC) and that the ATPase activity of HSC70 is involved in TGEV internalization during clathrin-mediated endocytosis (CME). Together, our findings clarified the specific mechanism underlying TGEV internalization and provided new insights into the early stages of the CoV life cycle.

## RESULTS

### HSC70 interacts with TGEV M protein.

**FIG 1:**
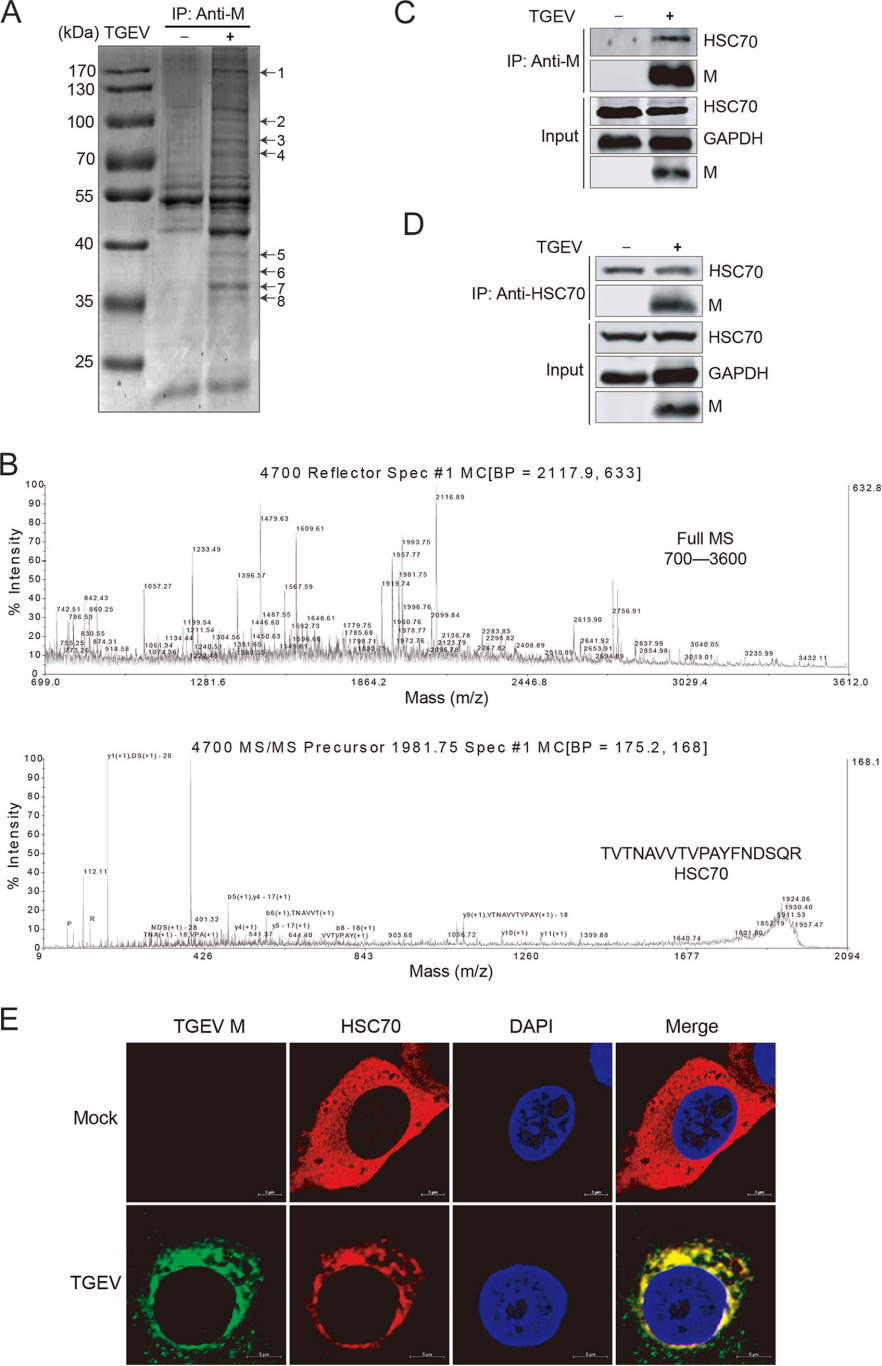
Identification of the cellular protein HSC70 by co-IP assays coupled with MS. (A) Screening of cellular membrane proteins interacting with TGEV M protein by anti-M antibody. SDS-PAGE analysis of co-IP assays. +, cell lysates of TGEV-infected porcine kidney 15 (PK-15) cells; −, lysates of mock-infected PK-15 cells. (B) MALDI-TOF MS spectra of peptides of HSC70. Protein bands were subjected to in-gel trypsin digestion, and the peptides were analyzed by MS. MALDI-TOF MS spectra of the identified cellular proteins associated with the TGEV M protein are shown. (C and D) Endogenous co-IP assays. PK-15 cells were infected (+) or mock infected (−) with TGEV. The cell lysates were subjected to co-IP analysis and analyzed by Western blotting with antibodies against HSC70 and M protein. (E) Subcellular localization of HSC70 (red) and TGEV M protein (green) by confocal microscopy. PK-15 cells infected with TGEV were fixed and subjected to immunofluorescence assays with primary antibodies of HSC70 and M protein and subsequent incubation with secondary antibodies labeled with Alexa Fluor 633 and Alexa Fluor 488. Nuclear staining was performed with DAPI (blue). Fluorescence images were acquired with a confocal microscope. Bars, 5 μm.

M protein is abundant in the envelopes of CoVs, and it plays key roles in the viral life cycle. We used an IP assay to identify M-interacting proteins as previously described, with monoclonal antibody (MAb) 1C3 directed against the TGEV M protein. Coomassie brilliant blue staining of SDS-PAGE gels containing the immunoprecipitated proteins detected eight more bands in the TGEV-infected cells coimmunoprecipitated with MAb 1C3 than in those in the control (Fig. 1A). These eight bands were manually excised from the gel for analysis by matrix-assisted laser desorption ionization–tandem time of flight mass spectrometry (MALDI-TOF MS) (Table 1). The protein bands were subjected to in-gel trypsin digestion, the peptides were analyzed by MS, and band 4 was identified as HSC70 (Fig. 1B).

**TABLE 1:** Cell proteins identified by MALDI-TOF MS

Band no.^*a*^	Protein name	Accession no.^*b*^	Mass (kDa)	No. of queries matched^*c*^	Protein score^*d*^	Peptide identified^*e*^
1	Clathrin heavy chain	gi|224492556	191.5	5	208	K.LLLPWLEAR.I K.VGYTPDWIFLLR. R.TSIDAYDNFDNISLAQR.L K.SVNESLNNLFITEEDYQALR.T R.LAELEEFINGPNNAHIQQVGDR.C
2	Moesin	gi|127236	67.6	2	61	K.IGFPWSEIR.N K.APDFVFYAPR.L
3	78-kDa glucose-regulated protein	gi|350579657	72.9	4	220	K.KSDIDEIVLVGGSTR.I K.VTHAVVTVPAYFNDAQR.Q K.DNHLLGTFDLTGIPPAPR.G R.IEIESFYEGEDFSETLTR.A
4	Heat shock 70-kDa protein 8	gi|345441750	70.8	2	106	K.TVTNAVVTVPAYFNDSQR.Q K.QTQTFTTYSDNQPGVLIQVYEGER.A
5	Ribosomal protein SA	gi|71057496	32.9	1	80	R.FTPGTFTNQIQAAFR.E
6	Unidentified					
7	Annexin A2	gi|52631987	38.5	3	118	R.QDIAFAYQR.R K.GVDEVTIVNILTNR.S K.AYTNFDAERDALNIETAIK.T
8	Ribosomal protein L6	gi|56384243	32.2	2	69	K.AVPQLQGYLR.S R.ASITPGTILIILTGR.H

a Protein band number as shown in Fig. 1A.

b Accession numbers according to the NCBI nr database.

c The number of peptides identified by MALDI-TOF MS was given by MASCOT.

d Proteins with scores greater than 59 were considered successfully identified (*P* < 0.05).

e The peptides identified by MALDI-TOF MS with statistically significant ion scores (confidence interval > 95%). Typical cleavage is performed at the C-terminal of amino acids K or R, with the cleavage points separated by dots. The sequence between these two dots is the peptide identified.

Western blotting was performed to verify the proteins identified by MS. Figure 1C shows that HSC70 coprecipitated with the TGEV M protein in an IP assay when MAb directed against M protein was used, confirming the result of the MALDI-TOF MS analysis. To further verify the interaction between HSC70 and M protein, an antibody directed against HSC70 was used to precipitate the M protein in TGEV-infected PK-15 cells. Figure 1D shows that TGEV M protein coimmunoprecipitated with HSC70 when treated with an anti-HSC70 antibody.

Colocalization of HSC70 and M protein in PK-15 cells infected with TGEV was observed by confocal laser scanning microscopy. As shown in Fig. 1E, HSC70 labeled with Alex Fluor 633 goat anti-rat IgG colocalizes with M protein labeled with Alexa Fluor 488 rabbit anti-mouse IgG. Taken together, these results indicated that HSC70 interacted with the M protein.

### HSC70 interacts with the endodomain of M protein through its SBD.

**FIG 2:**
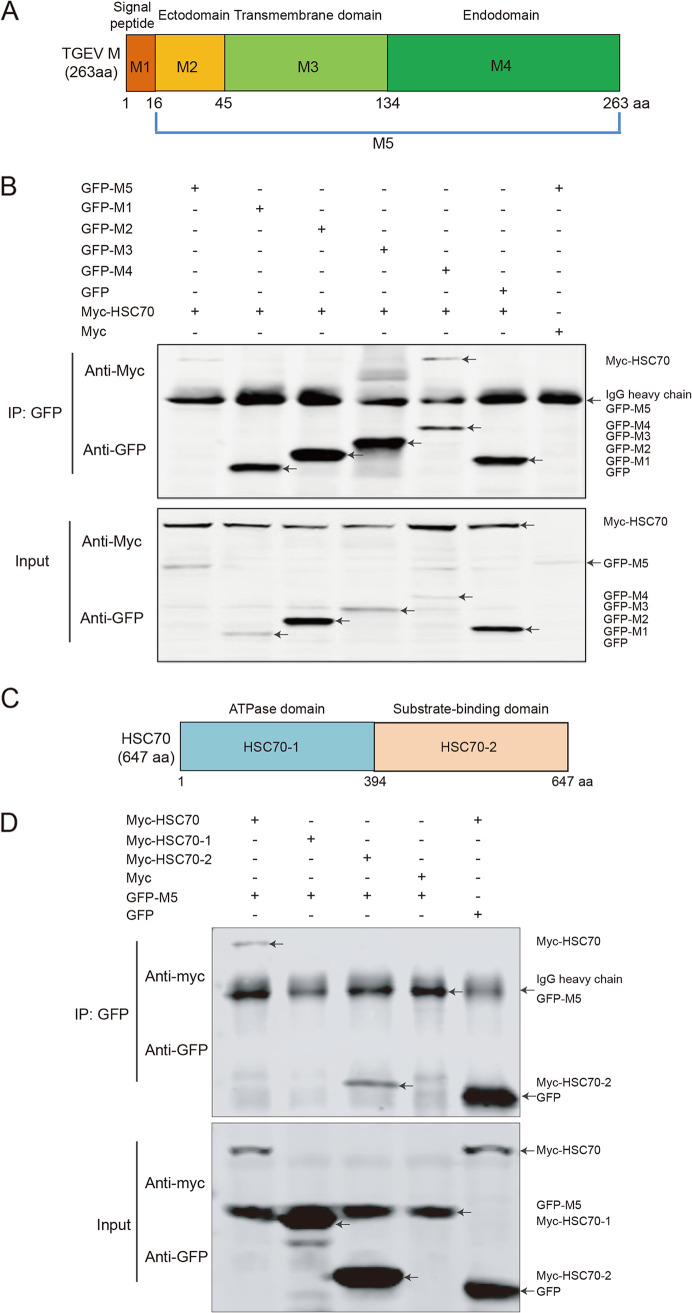
HSC70 interacts with the endodomain of the M protein through its SBD. (A) Schematic illustration of the full-length and truncated forms of GFP-M for the co-IP assays. The numbers indicate amino acid positions. (B) The plasmids for expression of GFP-M, four domains of M fusion protein, and Myc-HSC70 fusion protein were coexpressed in HEK293T cells. The protein complexes were immunoprecipitated with anti-GFP antibodies and analyzed by Western blotting with antibodies against Myc and GFP. (C) Schematic illustration of the full-length protein and two domains of HSC70 for co-IP assays. The numbers indicate amino acid positions. (D) The plasmids of Myc-HSC70, Myc-HSC70-1, Myc-HSC70-2, and GFP-M5 were coexpressed in HEK293T cells. The protein complexes were immunoprecipitated with anti-GFP antibodies and analyzed by Western blotting with anti-Myc and anti-GFP antibodies. Myc and GFP protein served as negative controls.

The M protein is divided into three major regions: the ectodomain, the transmembrane domain, and the endodomain (11). The signal peptide region of the M protein is located at aa 1 to 16. To determine the key region of M protein that interacts with the HSC70 protein, we constructed five plasmids to express distinct green fluorescent protein (GFP)-tagged domains of the recombinant M protein (Fig. 2A). Co-IP assays were carried out to detect the key domain of M protein. Briefly, Myc-HSC70 was coexpressed with GFP-M1, GFP-M2, GFP-M3, or GFP-M4 in HEK293T cells. The co-IP results showed that GFP-M4 (aa 135 to 263) coprecipitated with Myc-HSC70 after treatment with anti-GFP antibody (Fig. 2B), thus indicating that the endodomain of M protein was the essential region for the interaction.

HSC70 has two functional domains (26): the nucleotide-binding domain, encompassing a highly conserved ATPase domain, and the SBD, which binds peptide or protein substrates. After analysis of the structure of HSC70, we constructed two eukaryotic expression plasmids, Myc-HSC70-1 (aa 1 to 394) and Myc-HSC70-2 (aa 395 to 646) (Fig. 2C), to express the recombinant HSC70 domains. To identify the key region of HSC70 involved in the interaction with M protein, we cotransfected HEK293T cells with GFP-M5 and Myc-HSC70, Myc-HSC70-1, or Myc-HSC70-2. Figure 2D shows that the recombinant protein Myc-HSC70-2 coprecipitated with the GFP-M5 protein, thus indicating that the SBD of HSC70 was the essential region for the interaction. Further detailed studies are required to determine the exact amino acids of HSC70 responsible for the interaction.

### Knockout of HSC70 suppresses TGEV replication.

**FIG 3:**
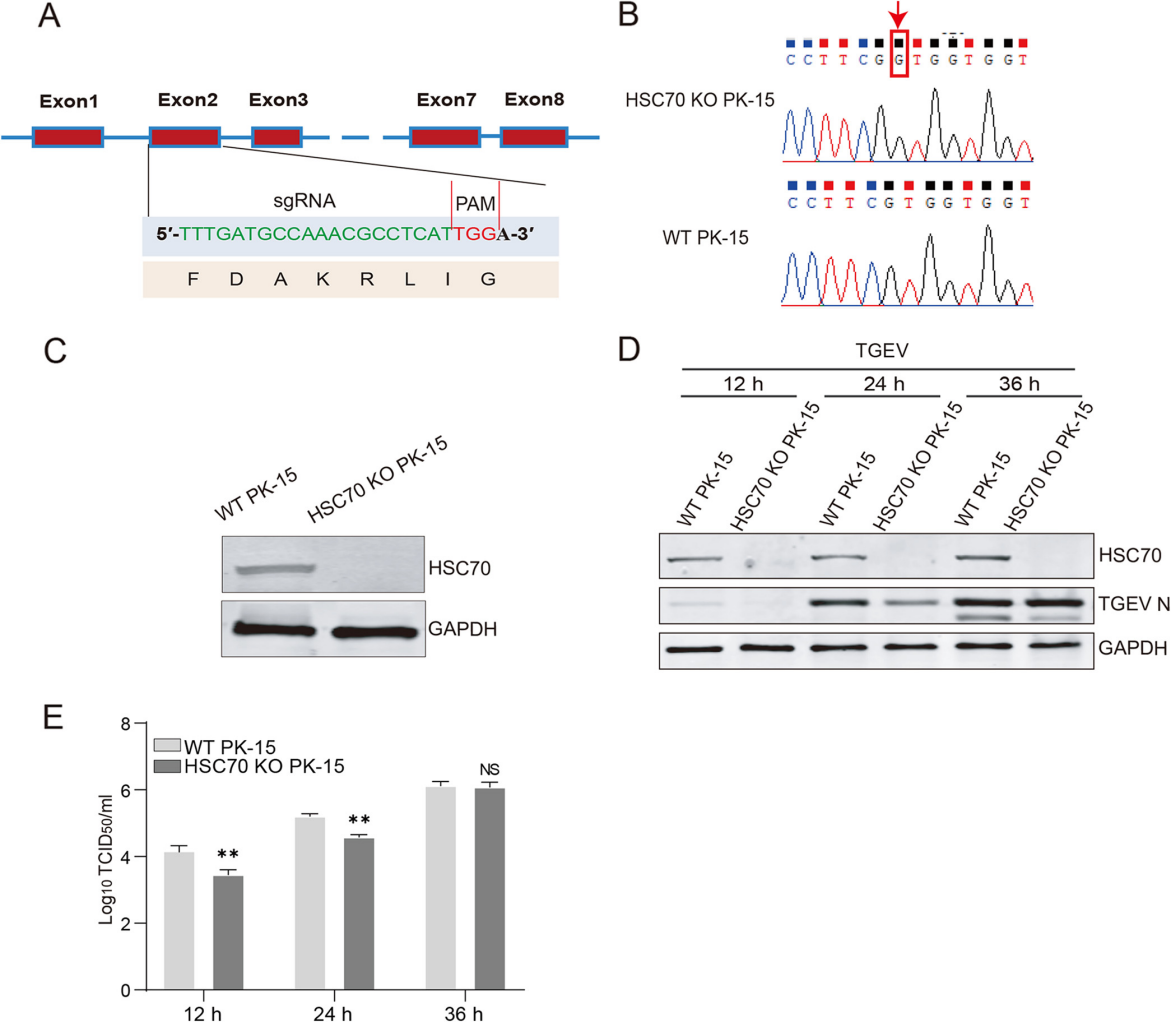
Knockout of HSC70 suppresses TGEV replication. (A) Schematic of the HSC70 knockout strategy; the Cas9/sgRNA target sites are indicated in green. (B and C) Confirmation of HSC70 knockout in a PK15 cell line using DNA sequencing and Western blotting. (D) Western blotting was performed to detect expression levels of N protein in WT PK-15 cells and HSC70 KO PK-15 cells at 12, 24, and 36 hpi. (E) Viral titers were examined. All experiments were conducted in triplicate. The error bars represent standard deviations calculated from three experiments. TCID_50_, 50% tissue culture infective dose. **, *P* < 0.01; NS, not significant (*P* > 0.05).

HSC70 has been demonstrated to participate in the life cycle of multiple viruses. To investigate the role of HSC70 in TGEV infection (27), we successfully generated an HSC70 knockout PK-15 cell line (HSC70 KO PK-15) using the CRISPR/Cas9 system. Briefly, the targeting regions for single guide RNA (sgRNA) located in exon 2 of HSC70 were selected through the CRISPR Design website (http://crispr.mit.edu) (Fig. 3A). The knockout efficiency was determined by DNA sequencing and Western blotting. DNA sequencing indicated that the deficiency was due to the presence of a single nucleotide insertion (Fig. 3B), impairing HSC70 protein translation from the 47-aa site and early termination of translation at 86 aa. Western blotting showed that HSC70 protein was completely knocked out (Fig. 3C), consistent with the results obtained from DNA sequencing.

To determine the role of HSC70 in TGEV replication, we infected wild-type (WT) PK-15 and HSC70 KO PK-15 cells with TGEV (multiplicity of infection [MOI] = 0.1) and separately collected the cell proteins and supernatants. The N protein of TGEV in the cells was detected with MAb 5E8 (28). As shown in Fig. 3D, the expression level of TGEV N protein was lower in HSC70 KO PK-15 cells than in WT PK-15 cells at 12 and 24 h postinfection (hpi). We also measured viral production, and knockout of HSC70 decreased viral production before 24 hpi (Fig. 3E). This difference was significant at 12 and 24 hpi. However, no significant difference was observed at 36 hpi, thus indicating that knockout of HSC70 suppressed TGEV replication in early stages. Together, these data suggested that downregulation of HSC70 suppressed TGEV replication in the early stage.

### HSC70 localizes on the cell surface.

**FIG 4:**
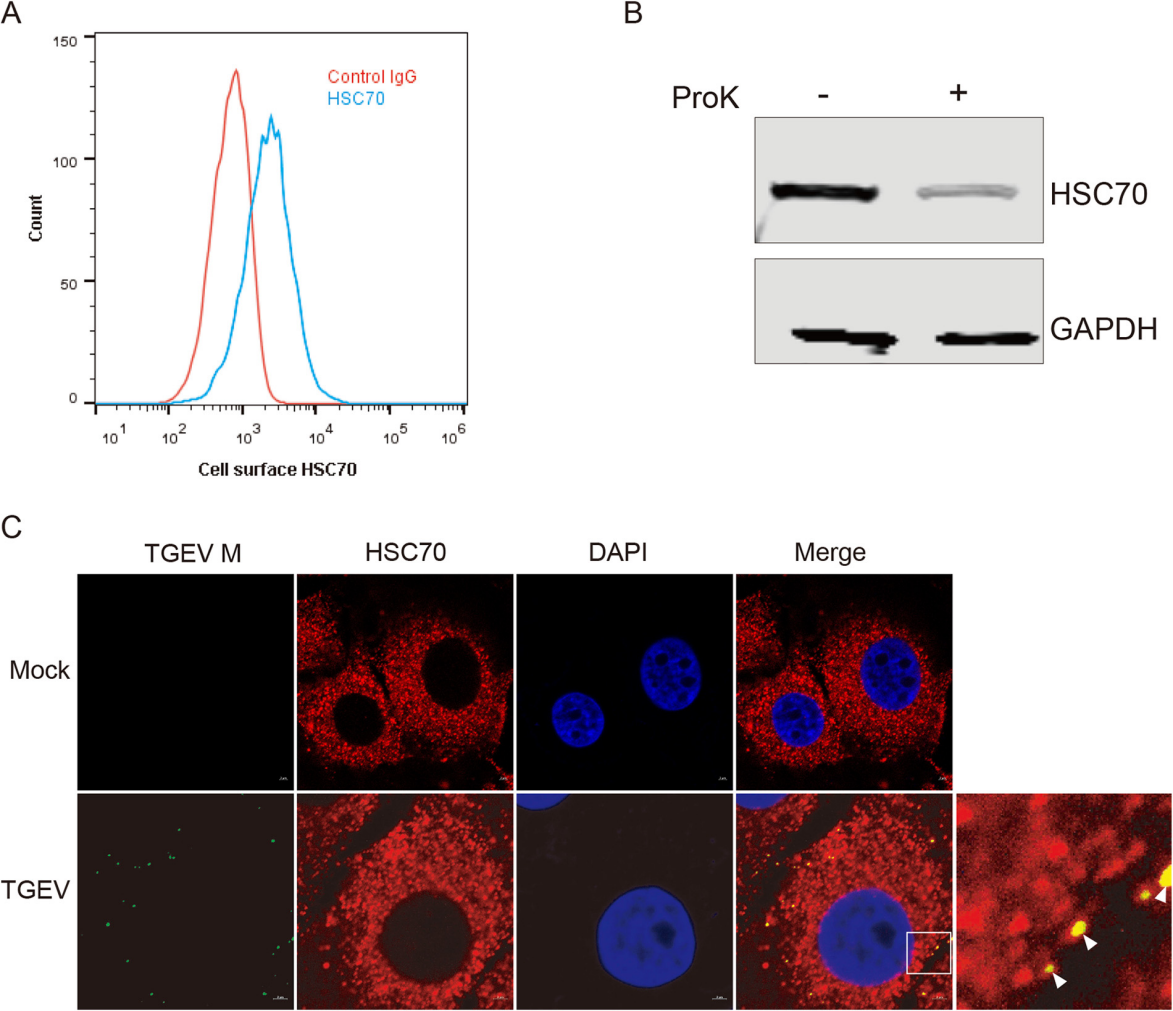
HSC70 localizes on the cell surface. Surface localization of HSC70 in PK-15 cells is shown. (A) Surface expression levels of HSC70 were detected by flow cytometry under unpermeabilized conditions in PK-15 cells. (B) PK-15 cells were treated (+) or not treated (−) with proteinase K (ProK) and harvested for Western blotting. (C) Immunofluorescence colocalization analysis of HSC70 (red) and M (green). TGEV-infected cells were incubated with antibodies against HSC70 and M. Nuclear staining was performed with DAPI (blue). Amplified colocalization spots within the merged image are indicated by small white boxes, and colocalization spots are indicated by white arrowheads. Bars, 2 μm.

To investigate whether HSC70 was present on the surfaces of cells, we examined cell surface expression of HSC70 by flow cytometry under unpermeabilized conditions. The results indicated that HSC70 was present on the cell surface in PK-15 cells (Fig. 4A). To further confirm that HSC70 localized on the cell surface, we performed protease protection assays. As shown in Fig. 4B, the addition of ProK resulted in lower HSC70 protein levels than those in control cells without ProK treatment.

We next examined the subcellular location of TGEV M protein and HSC70. The cells were incubated with an antibody against the TGEV M protein and then stained to visualize the viral particles. Immunofluorescence assays were carried out to confirm the interaction of cell surface HSC70 and M protein. We found that HSC70 and M colocalized on the cell surface of infected cells (Fig. 4C). Collectively, these results further confirmed that HSC70 is a cell surface protein that colocalizes with TGEV M on the cell membrane. Therefore, we hypothesized that HSC70 might be involved in early stages of TGEV infection.

### The interaction between HSC70 and M is involved in TGEV internalization.

**FIG 5:**
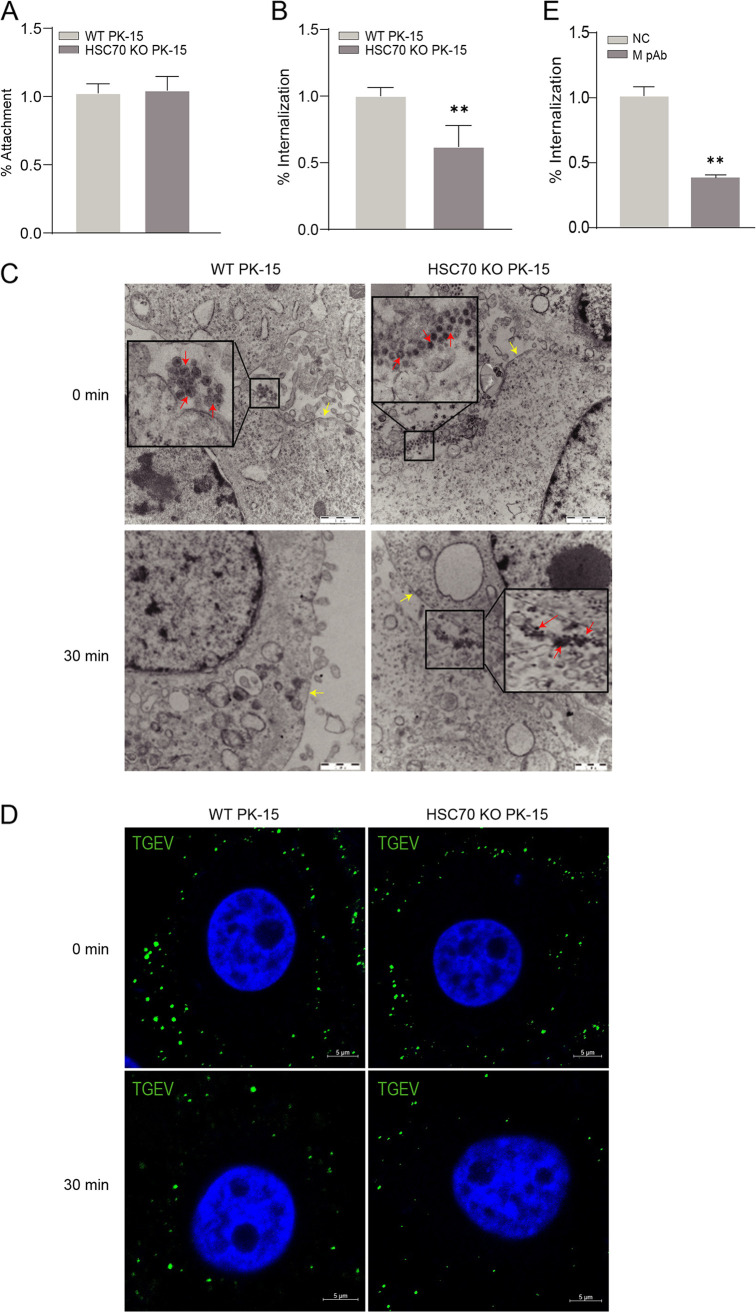
The interaction between HSC70 and M is involved in TGEV internalization. (A and B) Effect of HSC70 knockout on TGEV entry. The two cell lines were chilled and infected with TGEV (MOI = 1). After viral attachment at 4°C for 2 h, the cells were then washed with prechilled PBS three times. The cells were transferred to 37°C to facilitate viral internalization. At 30 min after infection, the cells were washed with prechilled PBS (pH 2.5) to remove noninternalized viruses. The virus RNA copies of attachment or internalization were determined by RT-qPCR. The percentage of RNA copies was calculated relative to the value obtained from untreated PK-15 cells. Statistical analysis: **, *P* < 0.01. All experiments were conducted in triplicate and produced similar results. (C) TEM analysis of TGEV internalization. The two cell lines were incubated with TGEV (MOI = 1) at 4°C. In attachment assays (0 min), one group was directly collected, fixed with 2.5% glutaraldehyde at 4°C for 24 h, and then fixed with 1.0% osmium tetroxide at 4°C for 2 h. Ultrathin sections of cells were observed under a TEM. The other group was subjected to internalization for 30 min, and the cells were collected for TEM analysis. Small black boxes represent amplified random visual fields containing virus, and small red arrows indicate viral particles. Yellow arrows indicate the cell membrane. Bars, 10 μm. (D) Time-dependent immunofluorescence analysis of TGEV internalization. The two cell lines were incubated with TGEV (MOI = 10) at 4°C. One group was directly fixed with 4% paraformaldehyde (0 min). The other group was subjected to internalization for 30 min, and both groups were incubated with antibodies against M for immunofluorescence analysis. Bars, 5 μm. (E) Blocking assays. PK-15 cells were cultured in 6-well plates, washed three times with PBS, and then infected with TGEV preincubated with PAb against M protein, denoted “M pAb,” or negative mouse serum (NC). Internalization assays were performed as described for panel B. Statistical analysis: **, *P* < 0.01. All experiments were conducted in triplicate and produced similar results.

The results described above showed that TGEV M interacted with HSC70 and that HSC70 influenced virus infection in the early stage. Therefore, we speculated that the interaction between M and HSC70 might be involved in the process of viral entry. To assess this hypothesis, we first performed viral attachment and internalization assays. Compared with that in WT PK-15 cells, there was no obvious difference in viral attachment (Fig. 5A) but the internalization efficiency was significantly reduced in HSC70 KO PK-15 cells (Fig. 5B). Second, transmission electron microscopy (TEM) was used to monitor the TGEV internalization process. As shown in Fig. 5C, many viral particles attached to the cell membrane in both WT PK-15 and HSC70 KO PK-15 cells, but after internalization for 30 min, almost no viral particles were observed on the WT PK-15 cells, whereas some viral particles were still observed on the HSC70 KO PK-15 cells, indicating that knockout of HSC70 reduced TGEV internalization.

To verify this result, we carried out a microscopy-based assay to monitor the internalization of TGEV. Briefly, HSC70 KO PK-15 and WT PK-15 cells were incubated with an antibody against TGEV M protein and then stained to visualize the viral particles. After internalization for 30 min, the fluorescence signal was mainly found on the cell membrane of HSC70 KO PK-15 cells, indicating that most of the virus remained on the cell surface. Conversely, the fluorescence signal was distributed in both the cell membrane and cytoplasm of WT PK-15 cells (Fig. 5D), thus indicating that the viral particles were restricted from entering the HSC70 knockout PK-15 cells.

In order to further validate that M protein was involved in TGEV internalization, we detected the effect of polyclonal anti-M serum (M PAb) on virus entry in PK-15 cells. First, we preincubated the virus with M PAb and then inoculated PK-15 cells with the mixture to perform internalization assays. We found that the internalization efficiency was reduced in the presence of M PAb (Fig. 5E). Together, these results suggested that HSC70 was involved in TGEV internalization and that blocking the interaction between M and HSC70 inhibited TGEV internalization.

### HSC70-mediated TGEV internalization is clathrin dependent.

**FIG 6:**
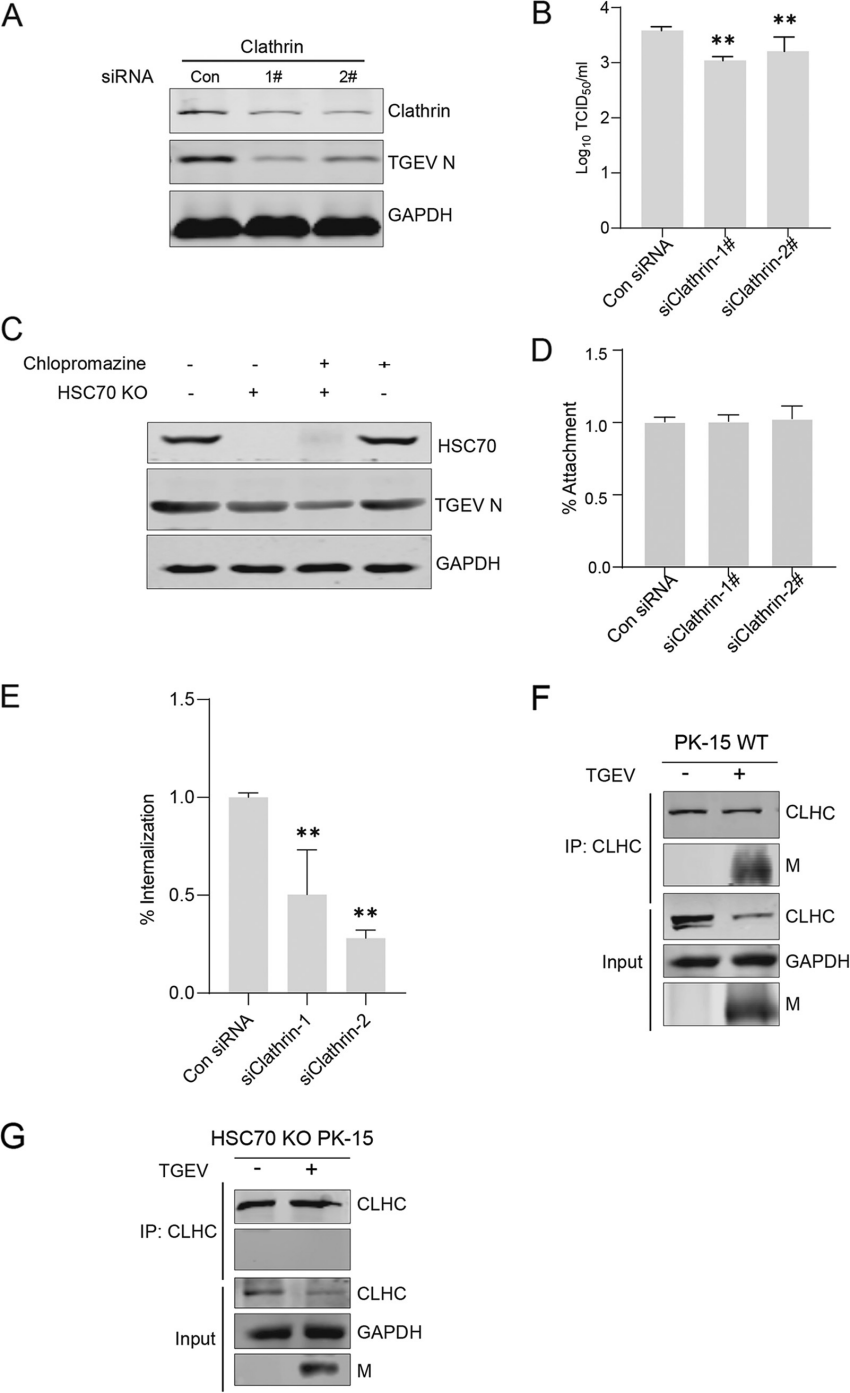
CME is involved in TGEV internalization in PK-15 cells. (A and B) Downregulation of clathrin decreased N protein levels and virus production. PK-15 cells were transfected with two siRNA duplexes targeting clathrin for 24 h. (A) Western blotting was performed to detect clathrin and N expression levels. (B) Virus titers. Statistical analysis: **, *P* < 0.01. All experiments were conducted in triplicate and produced similar results. (C) PK-15 cells were pretreated with chlorpromazine (10 μM) for 2 h. Cells were infected with TGEV (MOI = 0.1). At 12 hpi, cell lysates were collected for Western blotting. Dimethyl sulfoxide (DMSO) served as a negative control. All experiments were repeated at least three times and showed consistent results. (D and E) CME is involved in TGEV internalization. Downregulation of clathrin as described for panels A and B and attachment and internalization assays were performed. Viral RNA was collected for RT-qPCR analysis. The percentage of TGEV N copies was calculated relative to the value obtained from PK-15 cells treated with control (Con) siRNA. The data are presented as the mean and standard deviation (error bars) of three samples per group (**, *P* < 0.01). All experiments were repeated at least three times and showed consistent results. (F and G) Endogenous co-IP assays were performed to examine the interaction of CLHC and M. WT PK-15 cells and HSC70 KO PK-15 cells were infected (+) or mock infected (−) with TGEV, and the cell lysates were collected and subjected to co-IP analysis with anti-CLHC antibody. The IP and input complexes were analyzed by Western blotting with anti-CLHC and anti-M antibodies.

Several M-interacting candidate proteins were identified by MS (Table 1). Among them, clathrin was selected for subsequent studies owing to its prominent roles during TGEV infection. CME is the most commonly involved mechanism in the internalization of multiple viruses. A previous report has shown that CME is associated with TGEV internalization in swine testis cells (29). To further confirm the function of CME in TGEV entry into PK-15 cells, we examined TGEV infection in the presence of two small interfering RNAs (siRNAs) that targeted clathrin mRNA. The knockdown efficiency was confirmed by Western blotting, resulting in reduced levels of TGEV N expression (Fig. 6A) and viral production (Fig. 6B). Chlorpromazine, which inhibits the assembly of clathrin triskelions, was employed to suppress the CME pathway. The effect of chlorpromazine on TGEV internalization was quantified by Western blotting. As shown in Fig. 6C, after chlorpromazine treatment, the expression of TGEV N proteins was reduced in infected PK-15 cells, suggesting that CME was involved in TGEV internalization in PK-15 cells.

To further confirm the function of clathrin in TGEV infection and the TGEV life cycle, we performed attachment and internalization assays. We found that knockdown of clathrin had no obvious effect on TGEV attachment (Fig. 6D) but reduced the efficiency of TGEV internalization (Fig. 6E). Co-IP assays were performed to confirm the interaction of CLHC and M in WT PK-15 cells and HSC70 KO PK-15 cells, respectively. As shown in Fig. 6F and G, CLHC coprecipitated with M in WT PK-15 cells. However, the interaction between CLHC and M was not found in HSC70 KO PK-15 cells. Taken together, these results strongly indicated that M protein interacts with HSC70, thus directing TGEV internalization through the CME pathway.

### The ATPase activity of HSC70 plays an important role in TGEV internalization through CME.

**FIG 7:**
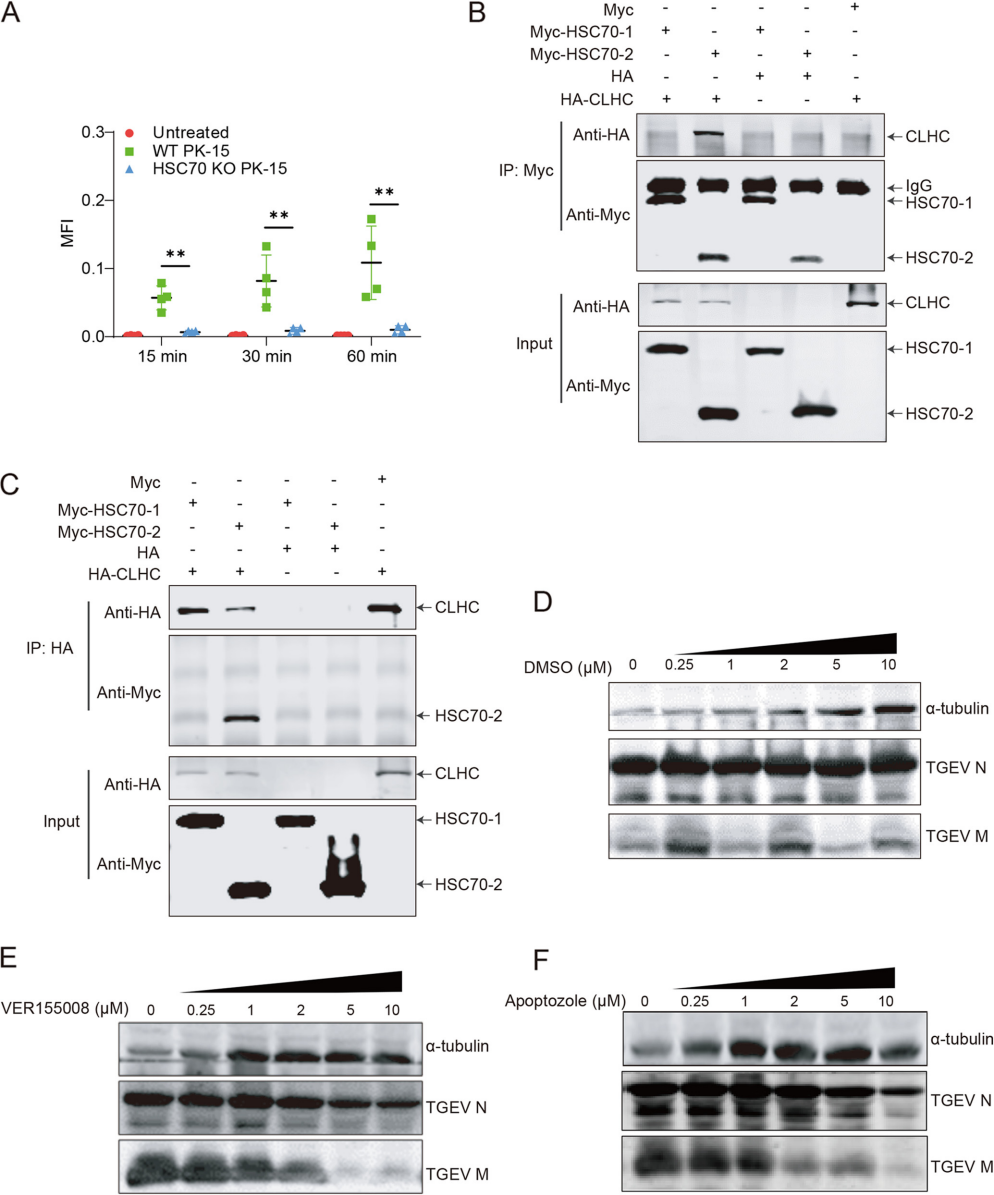
HSC70-mediated TGEV internalization is clathrin dependent. (A) Uptake of transferrin Alexa 488 by WT PK-15 and HSC70 KO PK-15 cells at 15, 30, and 60 min, quantified by flow cytometry. MFI, mean fluorescent intensity. (B and C) Co-IP assays were performed to examine the interaction of CLHC and HSC70. HA-tagged clathrin and Myc-tagged HSC70-1 and HSC70-2 were cotransfected into HEK293T cells. Co-IP assays were performed with anti-Myc and anti-HA antibodies and analyzed by Western blotting with anti-Myc and anti-HA antibodies. (D to F) Effects of HSC70 ATPase activity inhibition of TGEV infection. Inhibition of HSC70 by VER 155008 and apoptozole inhibited TGEV replication in PK-15 cells, and both effects were dose dependent at the indicated concentrations. Cell lysates were analyzed by Western blotting using anti-M and anti-N antibodies. DMSO served as a negative control.

Previous studies have shown that HSC70 is required for CME (30). Transferrin receptor is a well-studied cell surface transmembrane protein that undergoes CME (31). One of its principal roles is to acquire iron through CME but not through lipid raft-dependent endocytosis. Therefore, transferrin was used to evaluate the efficiency of CME in PK-15 cells in the internalization assay and flow cytometric quantification. Transferrin uptake was dramatically lower in HSC70 KO PK-15 cells than in WT PK-15 cells (Fig. 7A), thus suggesting that HSC70 was required in CME in PK-15 cells.

However, the molecular mechanism underlying HSC70’s involvement in CME remains unknown. To elucidate the molecular mechanism, co-IP assays were carried out to explore the correlation between HSC70 and clathrin. As shown in Fig. 7B and C, HSC70 interacted with CLHC, and the SBD of HSC70 was critical for this interaction. To further clarify the specific mechanisms underlying the involvement of HSC70 in CME, we used two HSC70 functional inhibitors, apoptozole and VER155008 (32, 33), to inhibit the ATPase activity of HSC70. Incubation of PK-15 cells with 10 μM inhibitors resulted in no significant toxicity. Next, we examined the effect on TGEV replication. As shown in Fig. 7D to F, both apoptozole and VER 155008 reduced the expression of TGEV N and M proteins in TGEV-infected PK-15 cells by inhibiting the disassembly of clathrin-coated vesicles, thus decreasing CME efficiency (34). These effects were dose dependent, thereby indicating that the ATPase activity of HSC70 plays a key role in TGEV internalization. Taken together, these results suggested that HSC70 facilitates TGEV internalization through CME.

## DISCUSSION

The CoV M protein is the most abundant protein in the virus and defines the shape of the viral envelope. It is also involved in virion assembly and release (35, 36). It has a small N-terminal glycosylated ectodomain and a much larger C-terminal endodomain, which extends 6 to 8 nm into the viral particle (37). However, studies have shown that a significant proportion of TGEV M protein (the carboxy-terminal domain) is exposed on the external surface of the virion (14). Generally, the protein domains exposed on the viral surface play important roles in the early stage of infection by binding to cell receptors, facilitating the process of virus-cell fusion, or by interacting with elements of the host immune system. The HCoV-NL63 M protein mediates viral attachment (15), indicating that the CoV M protein is involved in CoV entry, at least to some extent. In this study, we provided evidence that the TGEV M protein was involved in viral internalization in PK-15 cells. Remarkably, we found that TGEV M interacted with HSC70 to direct internalization via a CME pathway. The function of HSC70 in CoV infection was previously unknown. These findings subverted the findings of our previous studies that only the CoV S protein was responsible for inducing virus-cell membrane fusion (38, 39) and that M protein was the primary driver of the viral budding process (40).

The CoV M protein interacts with many other viral proteins and host cell proteins to regulate viral replication. During the assembly of the authentic virion, M interacts with itself, with the nucleocapsid protein N, with E, and with the S protein (41–43). Studies have shown that M protein forms a mushroom-shaped dimer, which can adopt two different conformations, allowing it to both bind to the nucleocapsid and promote membrane curvature (44, 45). In addition, based on the cryo-electron microscopy structure of the severe acute respiratory syndrome coronavirus 2 (SARS-CoV-2) M protein, the process of M protein-driven virus assembly has been proved (46). Interactions between the M and S proteins have been identified in infectious bronchitis virus (IBV), bovine CoV, and severe acute respiratory syndrome-related CoV (47–50). Identifying host cell proteins that interact with viral proteins is a critical step in understanding protein functions and viral replication.

Recently, affinity purification followed by MS has been widely used to identify protein-protein interactions (51). In the present study, a co-IP assay coupled with MS was used to precipitate the cellular membrane proteins that interact with the TGEV M protein. Seven cellular proteins, including HSC70 and clathrin, were successfully shown to interact with TGEV M protein. This is the first evidence of an interaction between HSC70 and the CoV M protein. Further detailed studies are needed to determine the exact amino acids responsible for the M-HSC70 interaction.

HSC70 is a member of HSP70 family. It has been conserved among different species during evolution (26). Previous research has attributed a large number of cellular functions to HSC70 (52). Topologically, HSC70 is a cytosolic protein, and increasing evidence indicates the presence of HSC70 on the cell surface (16). Our findings further support that HSC70 is present on the cell surface. Many cell surface molecules are involved in CoV infection, through various mechanisms, and HSC70 has many functions, including acting as a receptor for viral infection (53, 54). For example, HSC70 is involved in the binding of rotavirus, human T-cell lymphotropic virus type 1, and IBV to the cell surface (53–55). Marine medaka HSC70 also forms a complex with marine medaka HSP90ab1 and the nervous necrosis virus (NNV) capsid protein to facilitate viral entry (56). Therefore, we hypothesized that the interaction between HSC70 and M directed TGEV infection during its initial stages. First, we used MAb against M protein to precipitate HSC70, but N protein was not immunoprecipitated. We also used MAb against N protein to precipitate HSC70, and M protein was also immunoprecipitated. These results implied that there were several different modes of interaction between the M protein and HSC70 during the process of viral replication, including the M-HSC70 interaction and the complex interaction between HSC70 and the TGEV M and N proteins. Because M directly interacts with the coronavirus N protein (57), M/N/clathrin may bind HSC70 and form a complex. Given that N is a nucleocapsid protein, when viruses complete the uncoating process, N will be exposed; thus, M/N/clathrin may not bind HSC70 and form a complex under physiological conditions at the same time. Additional studies are warranted to confirm whether the interaction between N and HSC70 plays a role in the viral internalization process.

Coronaviruses use at least two pathways—membrane fusion and endocytosis—to enter host cells after binding the cellular receptor, in addition to a variety of other poorly characterized mechanisms (58). We found that after treatment with chlorpromazine, the virus still infected cells, thus indicating that the virus can enter host cells through different pathways. Furthermore, HSC70 knockout blocked transferrin endocytosis, thus suggesting that HSC70 is involved in the CME pathway in PK-15 cells.

Generally, the S-aminopeptidase N (APN) interaction is responsible for TGEV entry. However, our results confirmed that the M-HSC70 interaction also plays roles in TGEV internalization. One new question arises regarding the relationship between the S-APN and M-HSC70 pathways. Unlike some coronaviruses that do not yet have a well-studied receptor or have multiple receptors, the requirement for APN as the only cellular receptor for TGEV is well established (59). We demonstrated that knocking out HSC70 or using chemical inhibitors did not completely inhibit viral replication. One possibility is that M-HSC70-mediated entry is S-APN dependent and may play roles downstream of S-APN or facilitate the S-APN interaction on the cell membrane. Another possibility is that there are secondary effects of HSC70 that would, for example, assist in the folding of membrane proteins involved in S-mediated entry and produce a small but reproducible effect on binding, but we find that no difference in APN was observed at the mRNA level and a subcellular location. Interestingly, knockout of HSC70 also reduced porcine deltacoronavirus (PDCoV) infectivity, and the interactions between PDCoV M and HSC70 were also identified. Whether this is a general function or an adaptation of M protein in one species remains to be investigated.

CME is a classical endocytic pathway through which extracellular substances are transported into cells, and most enveloped and nonenveloped viruses enter host cells via this endocytic pathway (60). Here, CME was also found to be involved in TGEV replication in PK-15 cells, and knocking out HSC70 significantly decreased the internalization efficiency of CME. Critically, co-IP was observed between CLHC and M protein in WT PK-15 cells but not in HSC70 KO PK15 cells, thus indicating that M protein interacts with HSC70 and subsequently directs TGEV internalization through the CME pathway. Considering the presence of HSC70 on the cell surface, it may provide the possibility for other virion surface proteins to participate in virus internalization, such as S and E (25, 37). We have excluded the interaction between S, E, and HSC70 by co-IP assay, but it is difficult to separate M and S to evaluate the virus-HSC70 interaction. Future work will be necessary to further define the physiological roles of M-HSC70 during CME.

**FIG 8:**
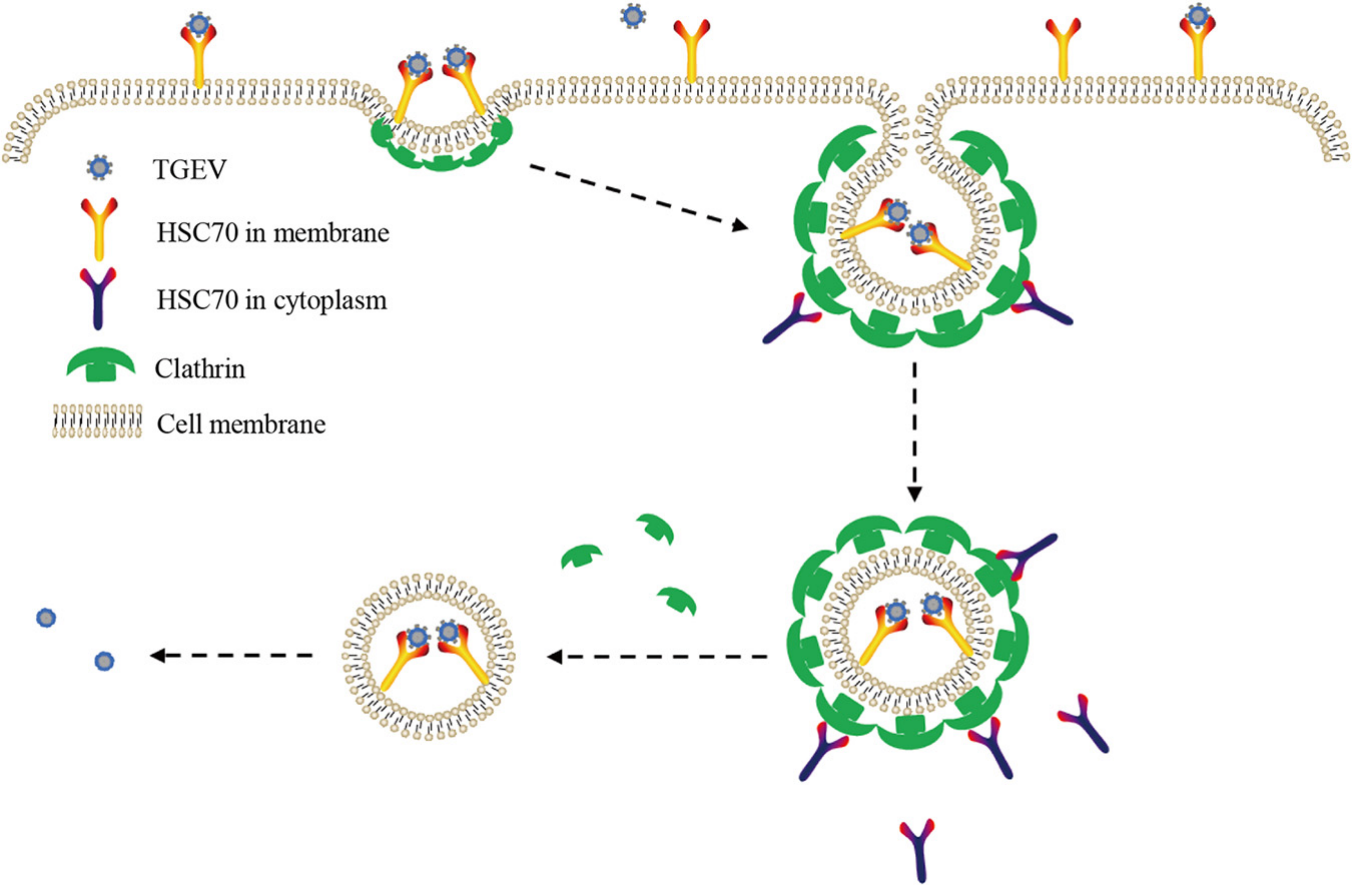
Model of the role of HSC70 in TGEV internalization. First, TGEV might attach to the PK-15 cell surface by binding to HSC70 through M protein or receptors. Then, the virus, HSC70, or receptor and clathrin form a protein complex. Finally, the virus is successfully internalized through the CME pathway, and the endocytic proteins are rapidly disassembled through the ATPase activity of HSC70 in the cytoplasm, thus facilitating TGEV release.

In conclusion, as shown in Fig. 8, HSC70 was identified as a novel host factor involved in TGEV internalization, and the TGEV M protein interacts with HSC70 in directing the invasion process of TGEV through CME. In this process, cell surface HSC70 plays important roles in TGEV internalization. Our data also showed that in addition to its role in viral assembly, the coronavirus M protein plays an important role in viral entry into cells. Therefore, this study highlights a novel mechanism of TGEV internalization into cells. These findings broaden our understanding of CoV infection during early stages.

## MATERIALS AND METHODS

### Cells, viruses, and antibodies.

PK-15 cells and human embryonic kidney (HEK293T) cells were maintained at 37°C in 5% CO_2_ in Dulbecco’s modified Eagle’s medium (DMEM; Sigma-Aldrich, catalog no. D6429) supplemented with 10% fetal bovine serum (FBS; HyClone, catalog no. SH30074.03). The TGEV infectious strain H165 (GenBank accession no. EU074218) used in this study was as described previously (61). PK-15 cells were seeded in 6-well plates; after reaching semiconfluence, the cells were infected with TGEV strain H165 (61). After virus adsorption for 1 h, the cells were washed three times and incubated with fresh DMEM.

Anti-Myc (M4439) and anti-hemagglutinin (HA) antibodies (M6908) were purchased form Sigma-Aldrich. Anti-GAPDH (glyceraldehyde-3-phosphate dehydrogenase) (ab8245), anti-HSC70 (ab1427, ab69558), and anti-CLHC (ab2731) antibodies were purchased from Abcam. Alexa Fluor 488-labeled rabbit anti-mouse IgG (H+L), Alexa Fluor 633-labeled goat anti-rat IgG (H+L) secondary, and Alexa Fluor 488-labeled goat anti-rat IgG (H+L) antibodies were purchased from Invitrogen Corporation. The MAbs against TGEV N (5E8) (28) and M protein (1C3) (62) and the PAbs against M protein were maintained in our laboratory.

### Construction of plasmids and transfection of cells.

Genes encoding viral M protein were amplified by reverse transcription–quantitative real-time PCR (RT-qPCR) and cloned into the pEGFP-C2 expression vector. The pEGFP-C2-M5 plasmid was constructed by insertion of the M-encoding fragment M5 (aa 49 to 789) into pEGFP-C2. Four partial TGEV M genes, corresponding to M protein aa 2 to 16 (nucleotides [nt] 4 to 48), aa 17 to 45 (nt 49 to 135), aa 46 to 134 (nt 136 to 402), and aa 135 to 262 (nt 403 to 789), were inserted individually into the pEGFP-C2 vector. Two partial HSC70 genes corresponding to HSC70 aa 2 to 394 (nt 4 to 1182) and aa 395 to 646 (nt 1183 to 1941) were inserted into the pCMV-Myc expression vector. The recombinant plasmids were designated pCMV-Myc-HSC70-1 (aa 2 to 394) and pCMV-Myc-HSC70-2 (aa 395 to 646), respectively.

HEK293T cells were transfected with plasmids with X-tremeGENE HP DNA transfection reagent (Roche, catalog no. 06366236001) according to the manufacturer’s instructions. Briefly, the cells were transfected with 100 μL of Opti-MEM (Gibco, catalog no. 2185847) containing 2 μg of the indicated plasmids and 2 μL of transfection reagent. At 6 h posttransfection (hpt), the transfection mixture was replaced with fresh DMEM containing 10% FBS and incubated for 36 h.

### Cell membrane proteins were immunoprecipitated with MAb against TGEV M protein.

PK-15 cells were infected with TGEV at an MOI of 0.1 and harvested at 24 hpi. The membrane proteins of the PK-15 cells were extracted with the ProteoExtract transmembrane protein extraction kit (Merck, catalog no. 71772).

Immunoprecipitation was performed as previously described (63). The cell membrane lysate (100 μg) was incubated with 1 μg of mouse IgG and 20 μL of protein A/G magnetic beads (Thermo Fisher, catalog no. 88803) for 2 h at 4°C with gentle agitation. The bead pellet was then discarded, and the supernatant was retained for the IP assay. The supernatant was mixed with an MAb directed against the TGEV M protein (1:300 dilution). After overnight incubation at 4°C, 20 μL of protein A/G magnetic beads was added and the mixture was incubated for another 2 h. After the sample was washed four times with lysis buffer, the immunoprecipitated proteins were boiled with SDS-PAGE sample loading buffer for 10 min and then analyzed by 12% SDS-PAGE. The cell membrane proteins of TGEV-uninfected PK-15 cells were used as the control.

### Protein identification with MALDI-TOF MS.

After electrophoresis, the protein bands were stained with PhastGel blue R (Sigma, catalog no. 3180753) and manually excised from the SDS-PAGE gel. MALDI-TOF MS was performed as previously described (64). MASCOT protein scores (based on a combination of the MS and MS/MS spectra) of >59 were considered statistically significant (*P* ≤ 0.05).

### Western blotting.

Cells were washed three times with phosphate-buffered saline (PBS) and lysed in lysis buffer for 30 min. Lysates were clarified by centrifugation at 12,000 × *g* at 4°C for 10 min. The lysates were subjected to SDS-PAGE and then transferred to nitrocellulose membranes. After the membranes were blotted, they were incubated with the indicated antibodies. The membranes were visualized with an Odyssey infrared imaging system (Odyssey CLX, USA).

### Immunoprecipitation of RNase A-treated HSC70.

The cell membrane protein lysate (100 μg) was incubated with 10 μL of RNase A (TaKaRa, catalog no. 2158), 1 μg rat IgG, and 20 μL of protein A/G magnetic beads for 2 h at 4°C with gentle agitation. The bead pellet was then discarded, and the supernatant was retained for the IP assay. The supernatant was incubated with 10 μL of RNase A and 1 μg of MAb directed against HSC70 overnight at 4°C with gentle agitation and then with 20 μL of protein A/G magnetic beads for another 2 h. The samples were washed four times with lysis buffer and analyzed by Western blotting.

### Immunofluorescence assay.

PK-15 cells were infected with TGEV. The cells were fixed with 4% paraformaldehyde. After permeabilization with 0.1% Triton X-100, the fixed cells were blocked with 5% bovine serum albumin and then incubated with antibodies directed against HSC70 or M protein overnight at 4°C. After being washed three times with PBS, the cells were incubated with Alexa Fluor 633-conjugated anti-rat IgG or Alexa Fluor 488 rabbit anti-mouse IgG antibodies. The cell nuclei were stained with 4′,6-diamidino-2-phenylindole (DAPI; Sigma-Aldrich). The cells were washed three times with PBS and observed with a confocal microscope (model LSM880; Zeiss, Thornwood, NY, USA).

### Co-IP assay.

HEK293T cells were cotransfected with the plasmids and then cultured for 24 h. The cells were lysed with Pierce IP lysis buffer (Thermo Fisher, catalog no. PI87787) on a rocker platform at 4°C for 30 min. The lysate was incubated with 1 μg of rabbit IgG and 20 μL of protein A/G magnetic beads for 2 h at 4°C with gentle agitation. The bead pellet was then discarded, and the supernatant was retained for the co-IP assay. The supernatant was incubated with 1.5 μL of rabbit PAb directed against GFP overnight at 4°C with gentle agitation and then with 20 μL of protein A/G magnetic beads for another 2 h. The samples were washed four times with lysis buffer and analyzed by Western blotting as mentioned above.

### RT-qPCR.

A QIAamp viral RNA minikit (Qiagen, catalog no. 52906) was used to extract the viral RNA from cells or the cell culture medium of PK-15 cells infected with TGEV, according to the manufacturer’s protocol. RNA was reverse transcribed into cDNA with a One Step PrimeScript RT-PCR kit (TaKaRa, catalog no. RR047A) according to the manufacturer’s instructions. Viral RNA copies were detected with specific primers (TGEV-N-F, 5′-TTTTGTTTGGAAGCTATTGGACT-3′; TGEV-N-R, 5′- CCTTTGGCAAGTGGTATTTGTG-3′). RT-qPCR was performed with an Mx3005P QPCR system (Agilent Technologies, Santa Clara, CA, USA).

### Attachment and internalization assays.

The kinetics of TGEV attachment were analyzed by adding the virus (MOI = 1) to chilled cells and incubating the cells at 4°C for 2 h, allowing viral attachment but preventing its internalization. After incubation for 2 h, the PK-15 cells were washed three times with chilled DMEM to remove any unbound virus and then lysed. After the attachment assay, the cells were transferred to a 37°C incubator for the internalization assay. The cells were washed with chilled PBS (pH 2.5) at specific time points to remove any virions retained on the cell surface and then lysed to determine the internalized viral RNA copies with RT-qPCR.

### Knockdown by siRNA.

Based on the coding sequence of the clathrin gene in PK-15 cells, siRNA targeting clathrin mRNA (targeting sequence, 5′-CAGCUUGUAAGACUGGGCAGAUCAA-3′, 5′-CAGACUGGAUCUUCCUGCUGAGAAA-3′) and negative-control siRNA were designed and synthesized by Life Technologies. The siRNA transfection was performed with Lipofectamine RNAiMAX reagent (Invitrogen, catalog no. 13778030) according to the manufacturer’s instructions.

### Establishment of HSC70 knockout cell lines.

PK-15 cells were used to generate HSC70 gene knockout cells by using CRISPR-Cas9 technology. Briefly, the targeting regions for sgRNA (TTTGATGCCAAACGCCTCAT) located in exon 2 of HSC70 were selected using the CRISPR Design website (http://crispr.mit.edu). The primers were synthesized at Comate Bioscience Company Limited (Jilin, China). The oligonucleotide pairs for the target sequences were annealed, and the resulting fragments were then cloned into pSpCas9(BB)-2A-Puro (PX459) V2.0 (65) and transfected into PK-15 cells. At 24 hpt, PK-15 cells with CRISPR/Cas9-mediated HSC70 knockout were selected by culture in puromycin (4.5 μg/mL)-containing DMEM for at least 10 days. The positive cell clones were validated by Western blotting and DNA sequencing. The primers synthesized for DNA sequencing were as follows: F-HSC70 (sense), 5′-CACCGTCTTCGTCCAATGAGGCGTT-3′; R-HSC70 (antisense), 5′-AAACAACGCCTCATTGGACGAAGA C-3′.

### Fluorescent ligand uptake and measurement.

Cells were starved in DMEM for 3 h, rinsed with cold PBS, and then treated with Alexa Fluor 488-conjugated transferrin (Life Technologies) at a concentration of 2 μg/mL for 2 h at 4°C. The cells were washed with chilled PBS to remove any unbound ligand and then transferred to a 37°C incubator for various periods. The cells were washed with PBS (pH 2.5) to remove any noninternalized ligand. The fluorescent signal in the cells was measured by flow cytometry (BD Accuri C6 Plus).

### Drug treatments.

Apoptozole (Selleck, catalog no. S8365) and VER 155008 (APExBIO, catalog no. A4387) were used to inhibit the ATPase activity of HSC70 in this experiment. PK-15 cells were treated with different concentrations of apoptozole or VER 155008 for 2 h before TGEV infection. The cells were infected with TGEV at an MOI of 0.1 and incubated for another 24 h with an inhibitor. The cells were washed three times with PBS and analyzed by Western blotting.

Chlorpromazine was used to inhibit CME (66). PK-15 cells were treated with chlorpromazine (10 μΜ) for 2 h before TGEV infection. The cells were infected with TGEV at an MOI of 0.1 and incubated for 24 h with the inhibitor. The infected cells were washed three times with PBS, harvested, and analyzed by Western blotting.

### Blocking assay.

TEGV was incubated with an equal volume of PAb against TGEV M protein for 1 h at 37°C. Mixed samples were added to PK-15 cells, which were cultured in 6-well plates for 48 h; after semiconfluence was reached, internalization assays were performed. As a control, an identical assay was performed with negative mouse serum. RT-qPCR was performed to determine the internalized viral RNA copies.

### TEM analysis.

Attachment and internalization assays were performed in WT PK-15 and HSC70 knockout PK-15 cells, and viral particles were observed by TEM. Ccell samples were prepared for TEM as reported previously (67). Briefly, the cells were collected and fixed with 2.5% glutaraldehyde at 4°C for 24 h and then fixed with 1.0% osmium tetroxide at 4°C for 2 h. Ultrathin sections of the cells were observed under a Hitachi H-7650 TEM.

### Proteinase K protection assay.

PK-15 cells were seeded into 6-well culture plates for 24 h. Cells were washed with PBS three times and treated with 10 μg/mL of proteinase K (ProK) for 30 min at 4°C. The reaction was stopped by the addition of phenylmethylsulfonyl fluoride. Then cells were lysed with radioimmunoprecipitation assay lysis buffer at 4°C for 30 min, and cell lysates were resuspended in SDS loading buffer and analyzed by Western blotting.

### Flow cytometry.

PK-15 cells were seeded in 6-well plates for 24 h, and harvested cells were treated with 0.25% trypsin for flow cytometric analysis. The cells were fixed with 4% paraformaldehyde at room temperature for 15 min and then washed three times with fluorescence-activated cell sorter (FACS) wash buffer (PBS containing 2% fetal calf serum [FCS]), and after incubation for 1 h with antibody to HSC70 (Abcam, ab69558) or IgG isotype control antibody (Abcam, ab37361), the cells were washed and stained with goat anti-rat Alexa Fluor 488 (Thermo Fisher, A11006) for 1 h. All samples were analyzed using a FC500 flow cytometer (Beckman Coulter). Cell surface mean fluorescence density was measured and analyzed using FlowJo software (FlowJo LLC).

### Statistical analysis.

Data are presented as means ± standard deviations (SD). GraphPad Prism 8 (Graph Pad Software, Inc., San Diego, CA, USA) was used for all statistical analyses. An unpaired two-tailed Student *t* test was used to evaluate the differences between groups.
